# Supplementary material for: Genome-Wide Comparative Analysis of the Cytochrome P450 Monooxygenase Family in 19 Aphid Species and Their Expression Analysis in 4 Cereal Crop Aphids
Source: Int J Mol Sci. 2024 Jun 18;25(12):6668. doi: 10.3390/ijms25126668 (PMC11203792; doi:10.3390/ijms25126668)
Supplement: Supplementary file 1 [file ijms-25-06668-s001.zip › Supplementary Figures.pdf]

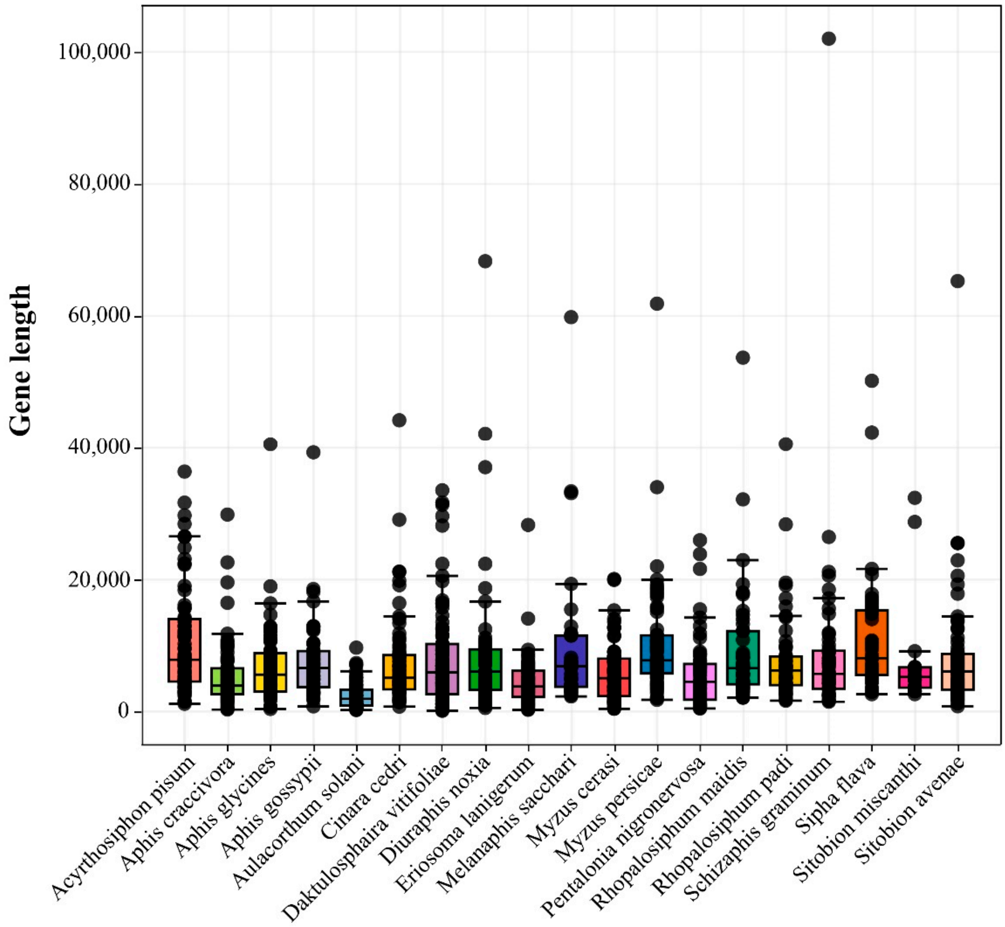

Figure S1. The gene length of all *CYP450* genes in 19 aphid species.

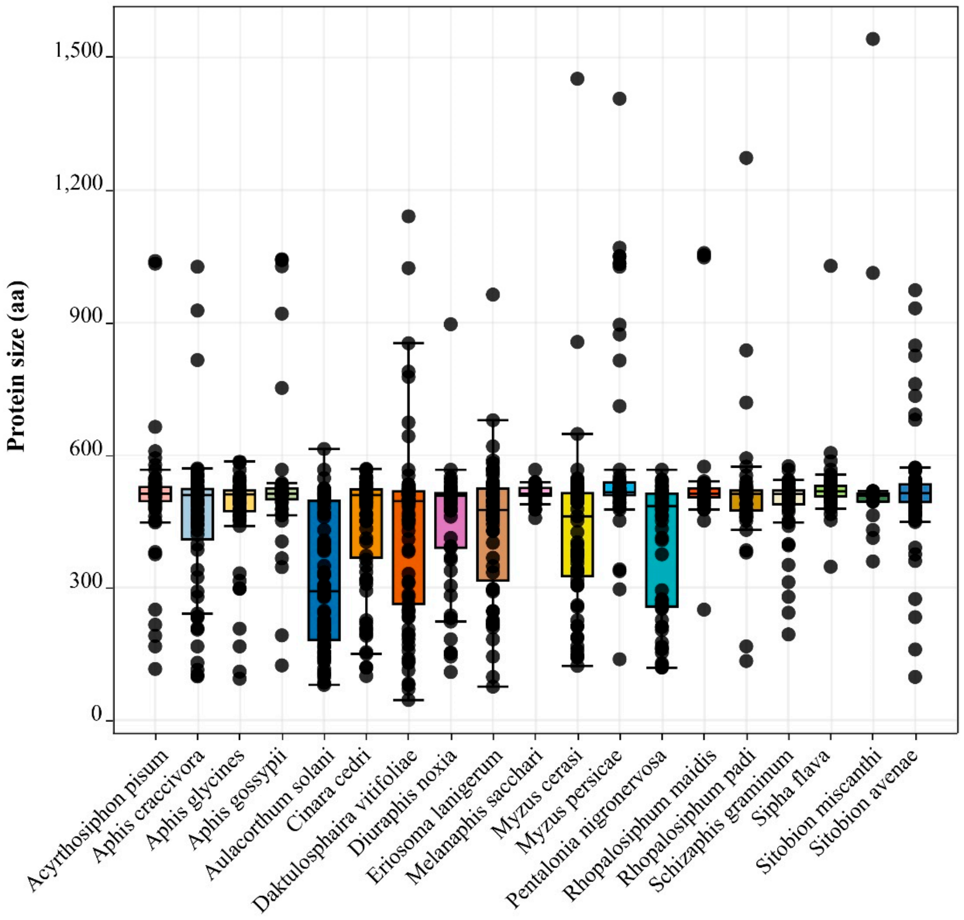

Figure S2. The predicted isoelectric points (pI) of all CYP450 proteins in 19 aphid species.

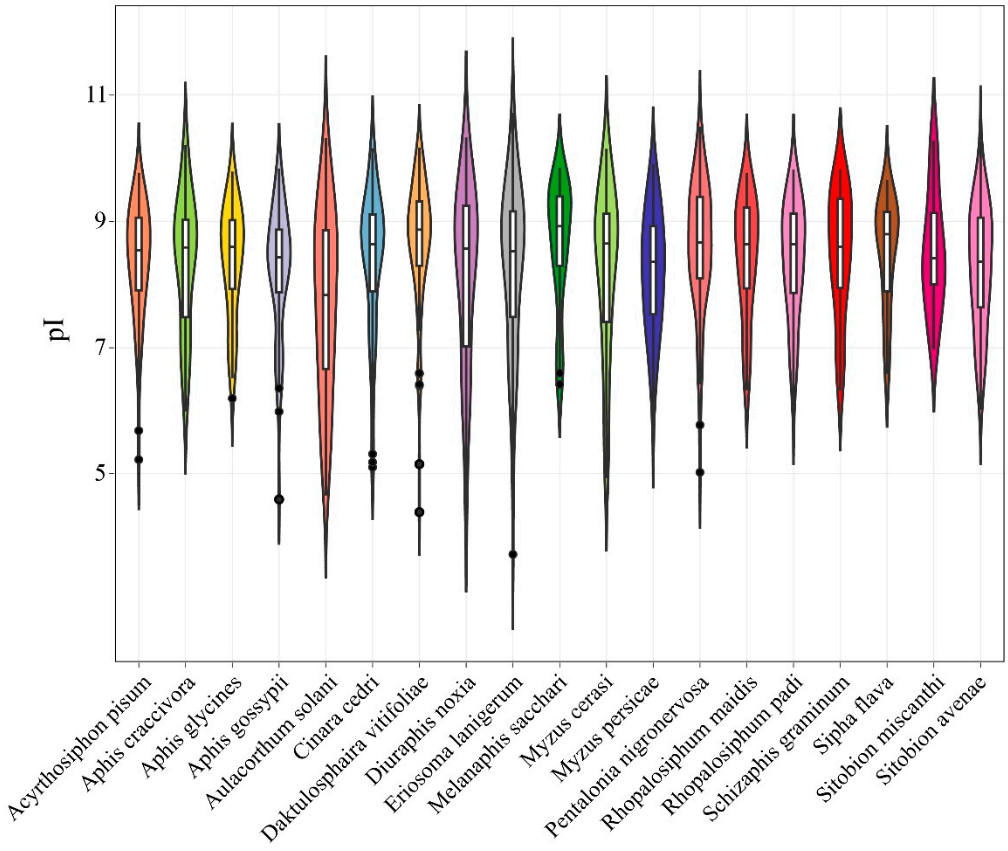

Figure S3. The predicted isoelectric points (pI) of all CYP450 proteins in 19 aphid species.

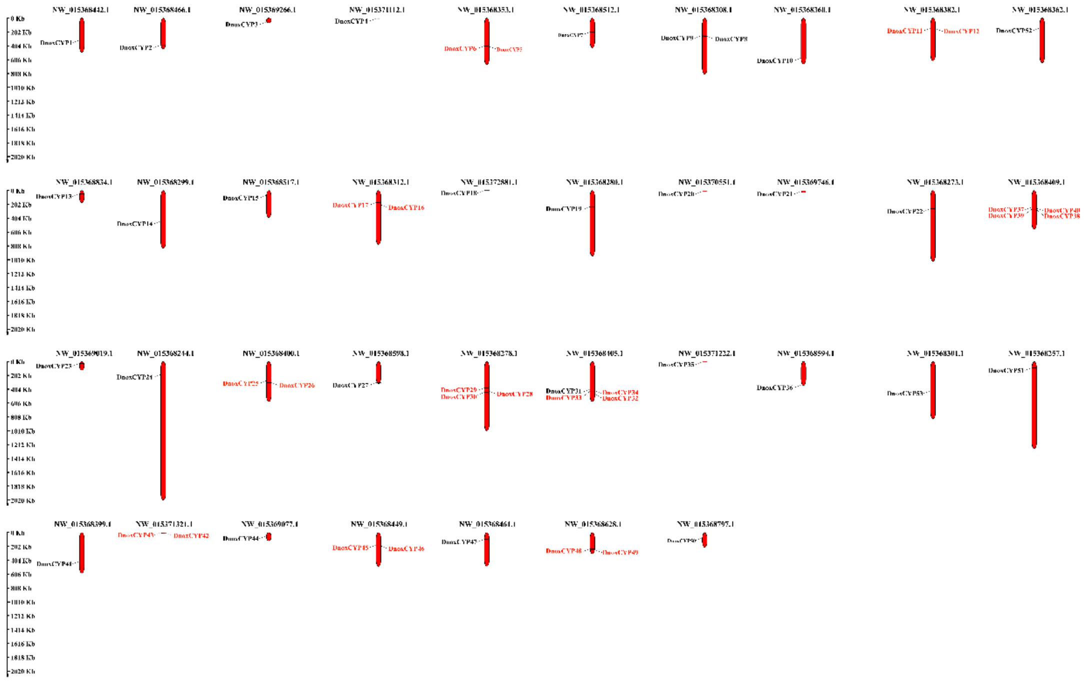

Figure S4. Distribution of the identified *CYP450* genes across the aphid genome of *Diuraphis noxia*. All scaffolds are drawn to scale based on their actual physical lengths. The gene pairs marked in red are tandem duplications.

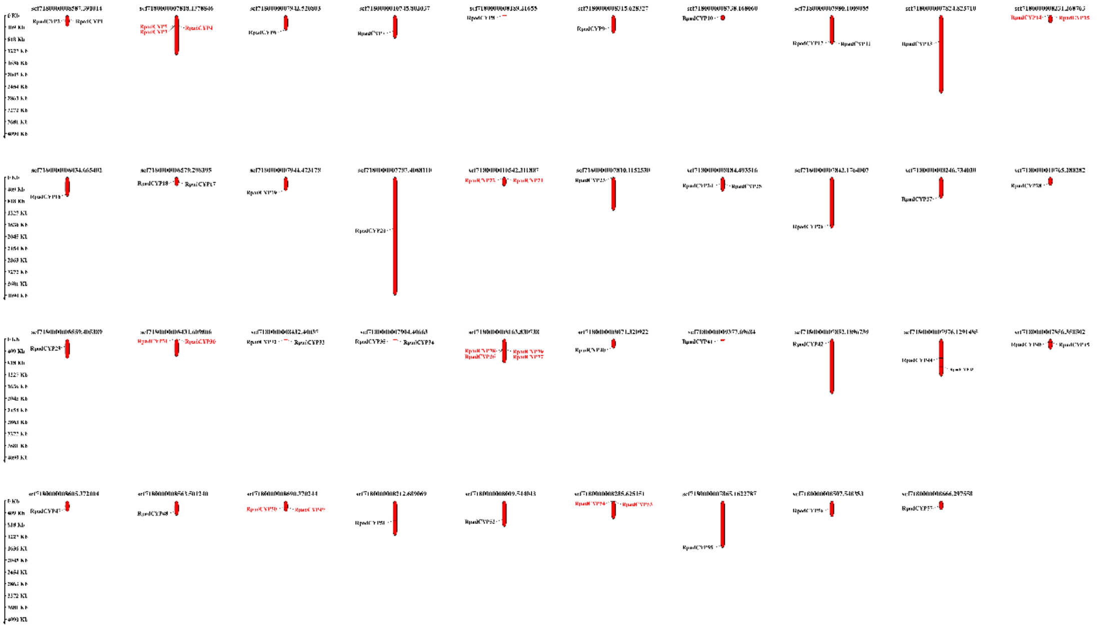

Figure S5. Distribution of the identified *CYP450* genes across the aphid genome of *Rhopalosiphum padi*. All scaffolds are drawn to scale based on their actual physical lengths. The gene pairs marked in red are tandem duplications.

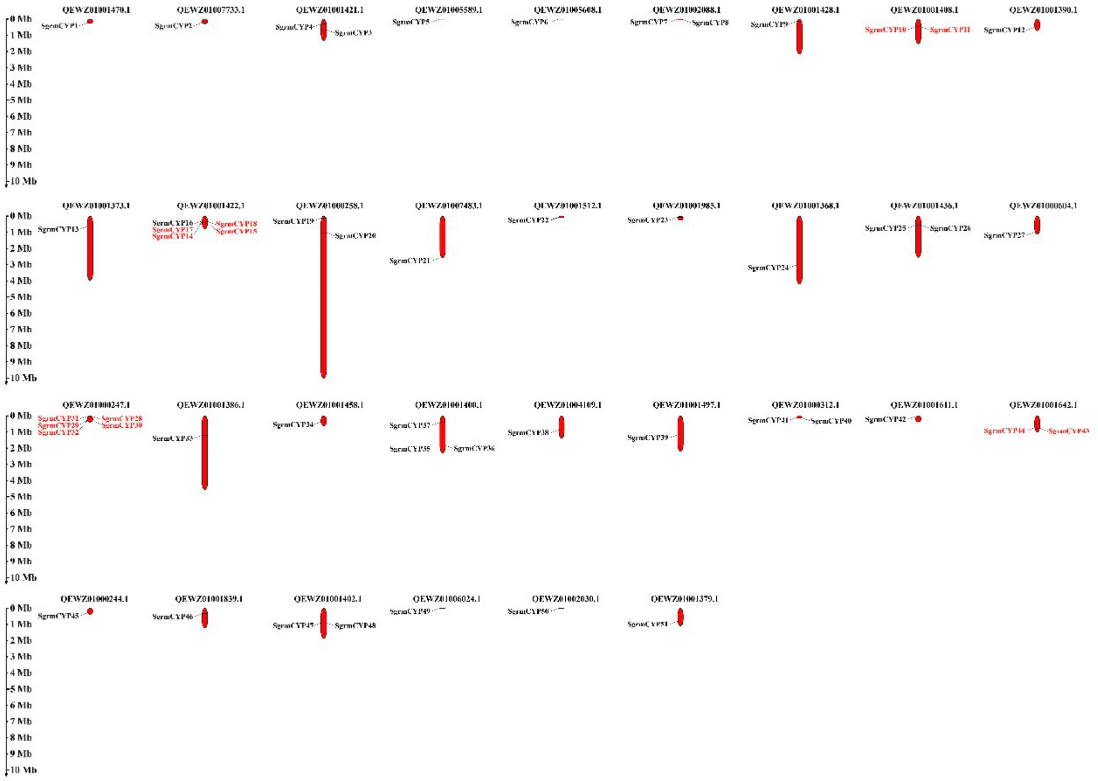

Figure S6. Distribution of the identified *CYP450* genes across the aphid genome of *Schizaphis graminum*. All scaffolds are drawn to scale based on their actual physical lengths. The gene pairs marked in red are tandem duplications.

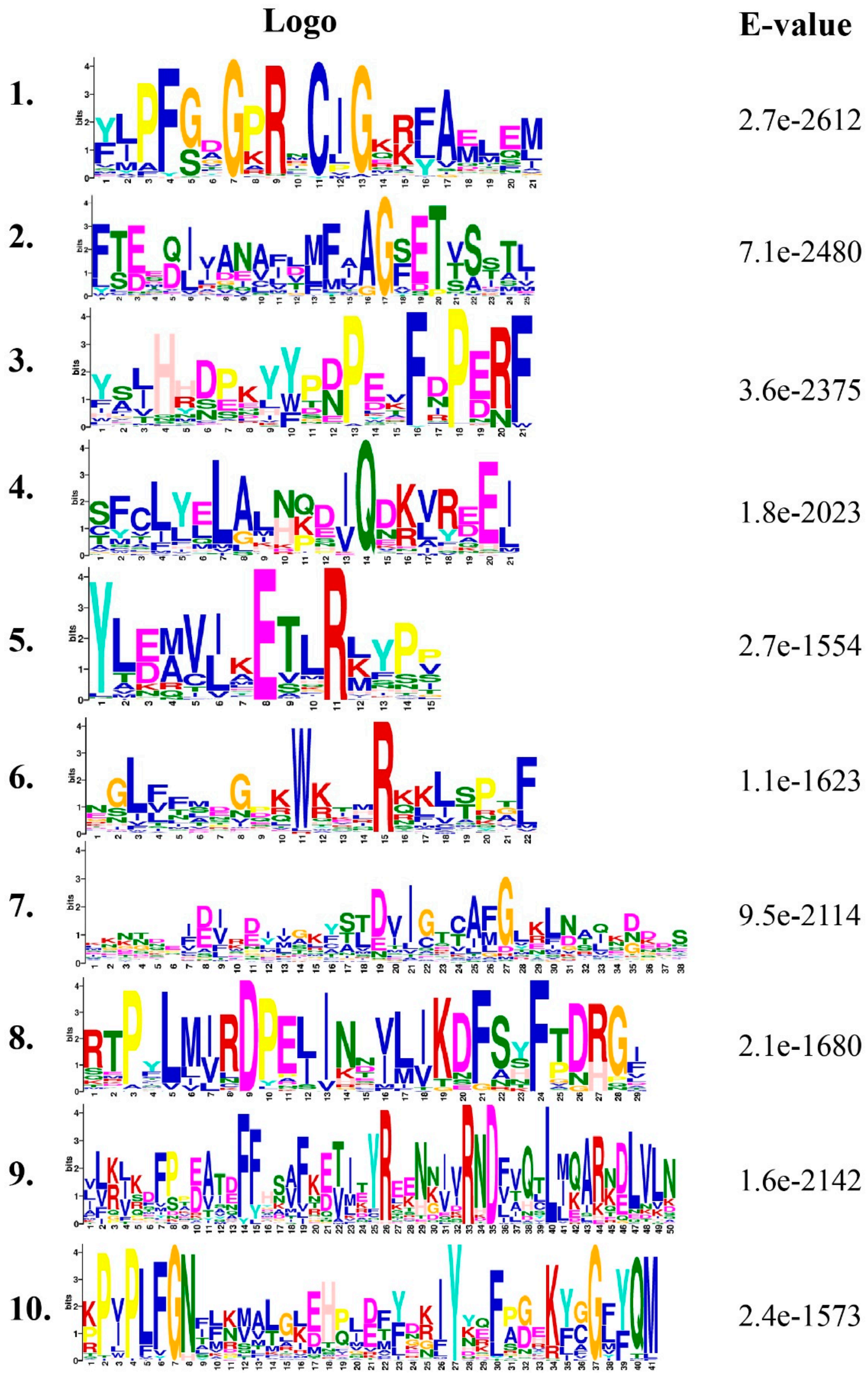

Figure S7. Conserved amino acid motif logos of identified 300 *CYP450* genes among cereal crop aphid species.

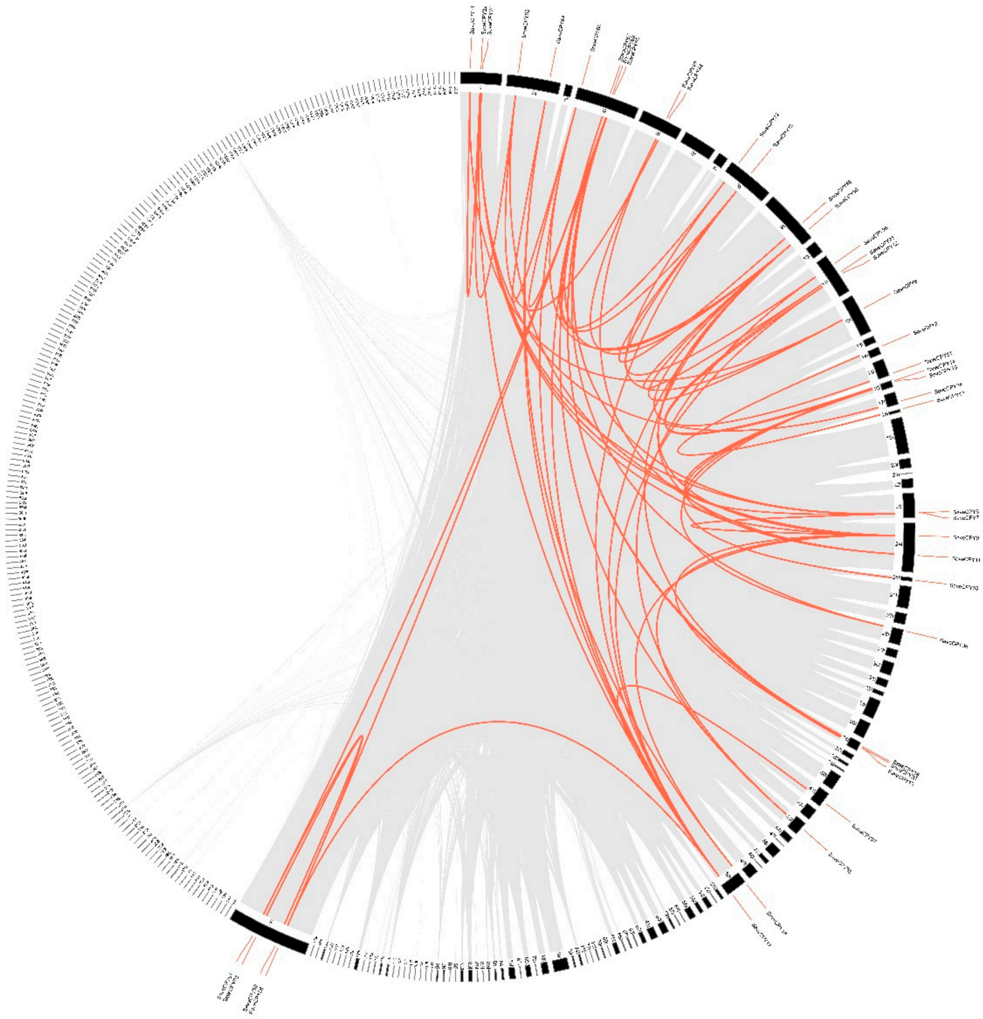

Figure S8. Duplication events of *CYP450* genes in the genome of *Sitobion avenae.* Black circos represent each scaffold of *Sitobion avenae*. Red lines represent duplicated pairs.

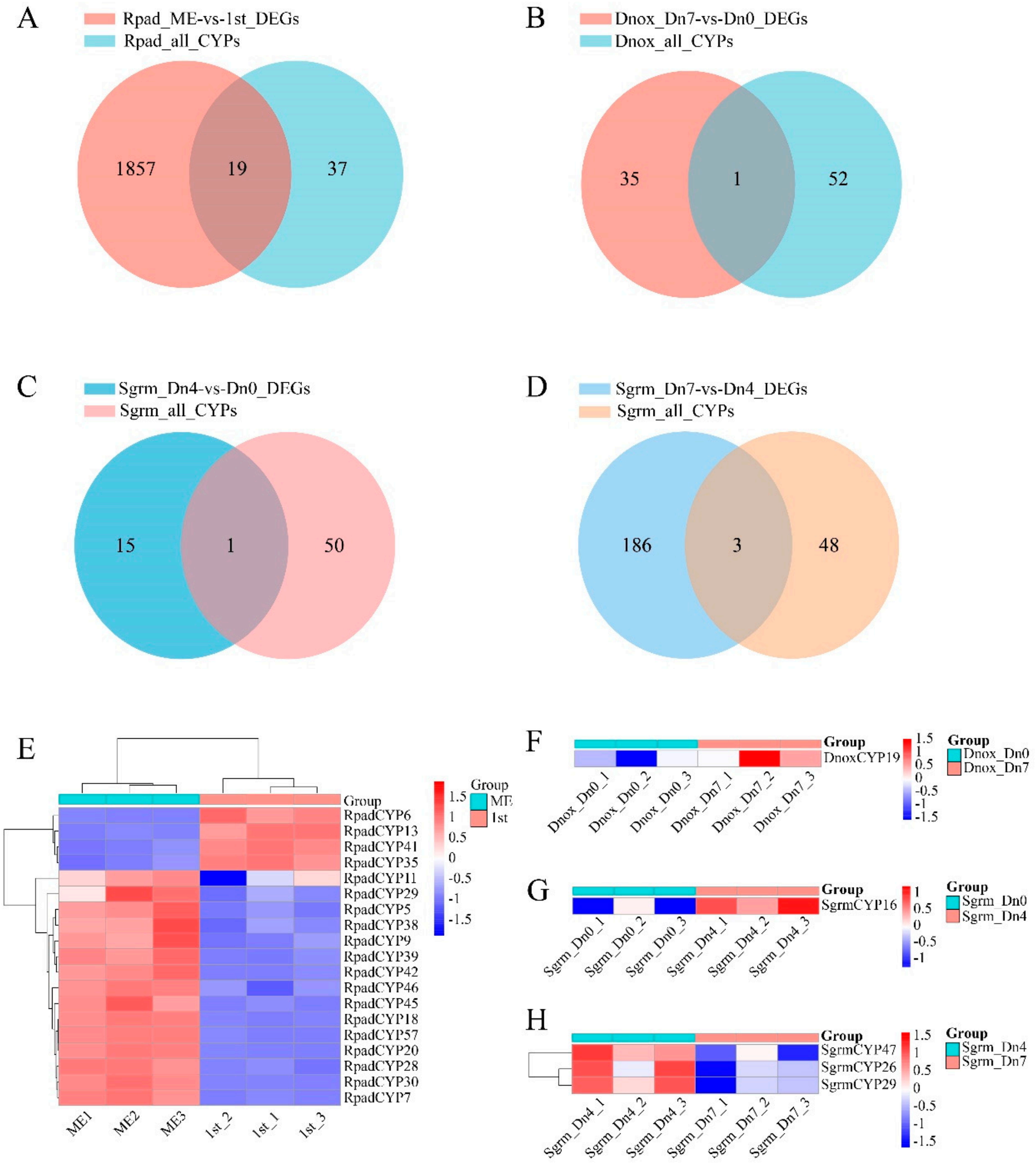

Figure S9. Differentially expressed *CYP450* genes of *Rhopalosiphum padi* (A), *Diuraphis noxia* (B) and *Schizaphis graminum* (C) aphids.

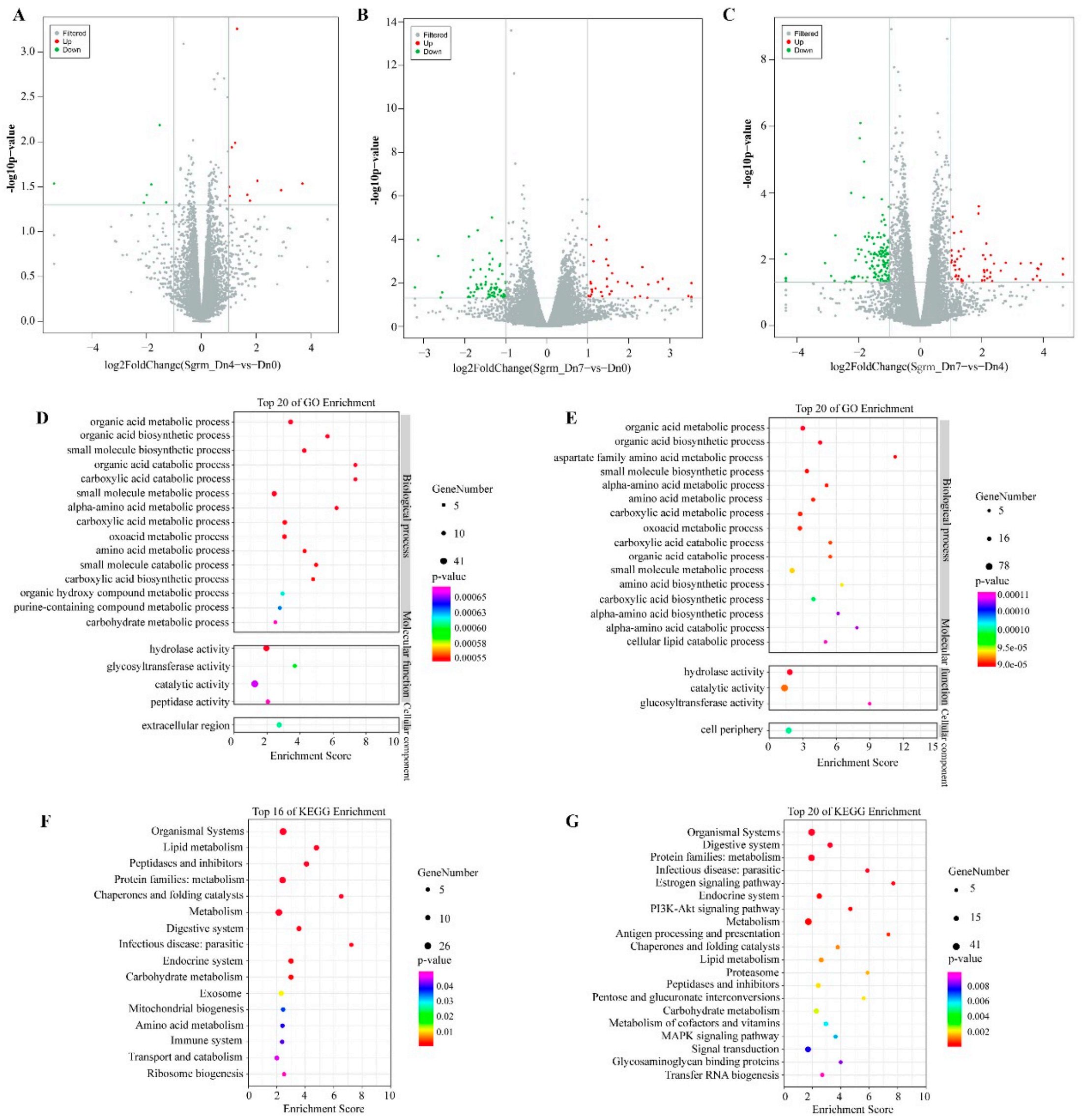

Figure S10. Transcriptomic overview of *Schizaphis graminum* fed on wheat plants containing different resistance genes *Dn0*, *Dn4* or *Dn7*. (A-C) Volcano plots of differentially expressed genes (DEGs) between different groups. (D) GO enrichment analysis of DEGs in the *Dn7* vs *Dn0* group. (E) GO enrichment analysis of DEGs in the *Dn7* vs *Dn4* group. (D) KEGG enrichment analysis of DEGs in the *Dn7* vs *Dn0* group. (E) KEGG enrichment analysis of DEGs in the *Dn7* vs *Dn4* group.

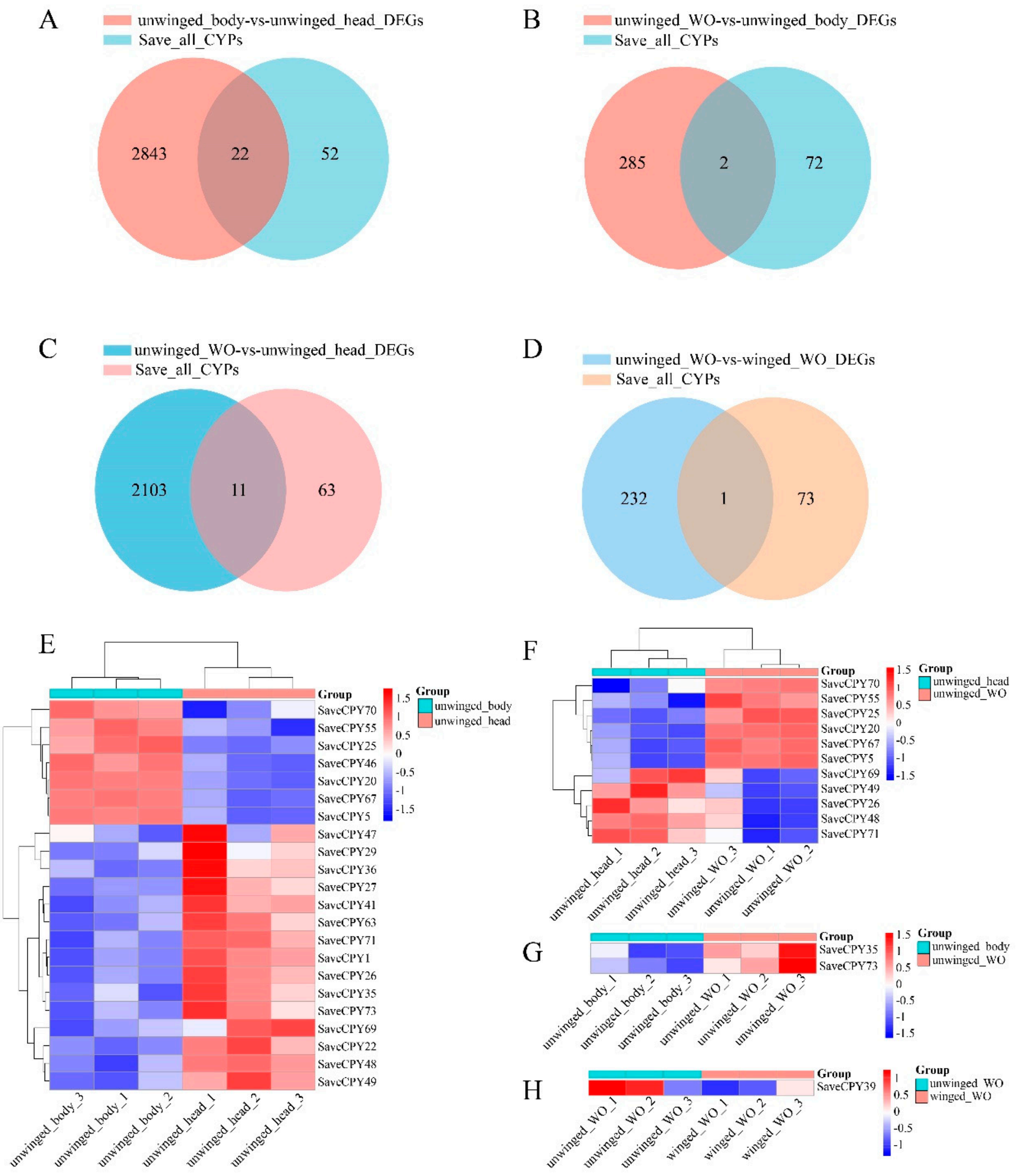

Figure S11. Differentially expressed *CYP450* genes of *Sitobion avenae* unwinged and winged aphids in different tissues.

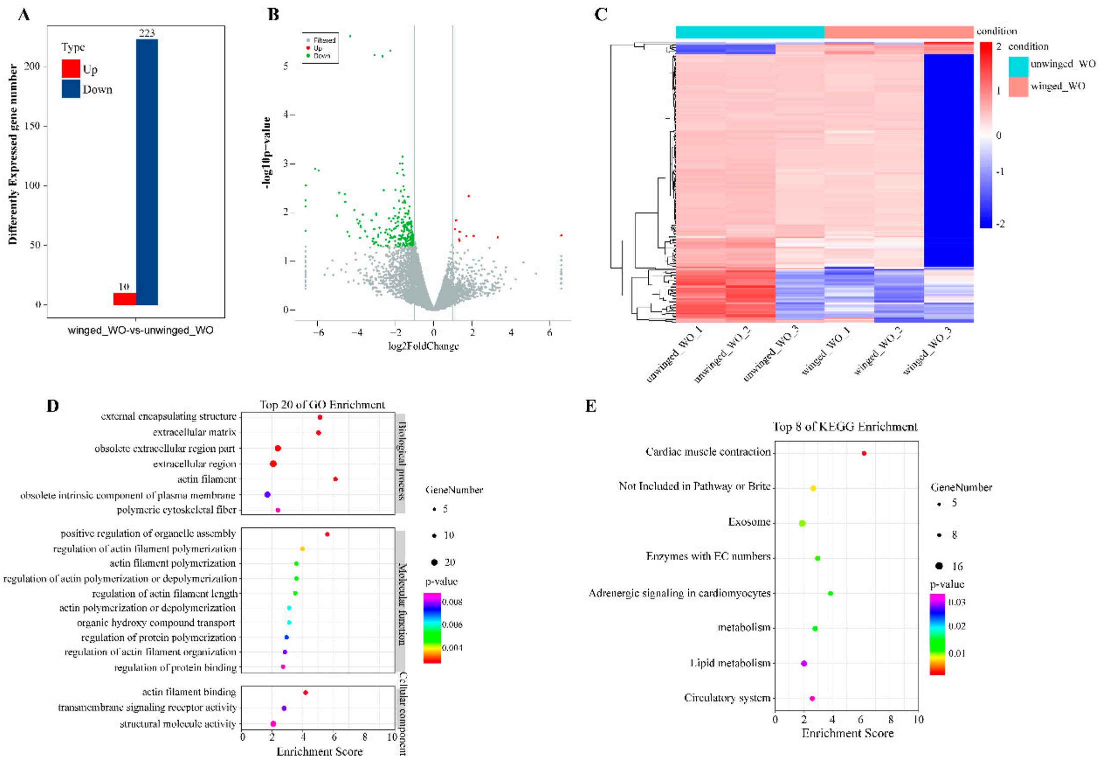

Figure S12. Transcriptomic overview of unwinged and winged *S. avenae* aphids. (A) Total number of significantly up- or downregulated genes. (B) Volcano plots of differentially expressed genes (DEGs) between unwinged and winged *S. avenae* aphids. (C) Expression patterns of differentially expressed genes (DEGs) between unwinged and winged *S. avenae* aphids. The colour scale represents Log2 expression values. The expression level is equal to the mean values and transform log 2 values. WO represents the whole organism of *S. avenae* aphids. (D) GO enrichment analysis of the DEGs between unwinged and winged *S. avenae* aphids. (E) KEGG enrichment analysis of the DEGs between unwinged and winged *S. avenae* aphids.

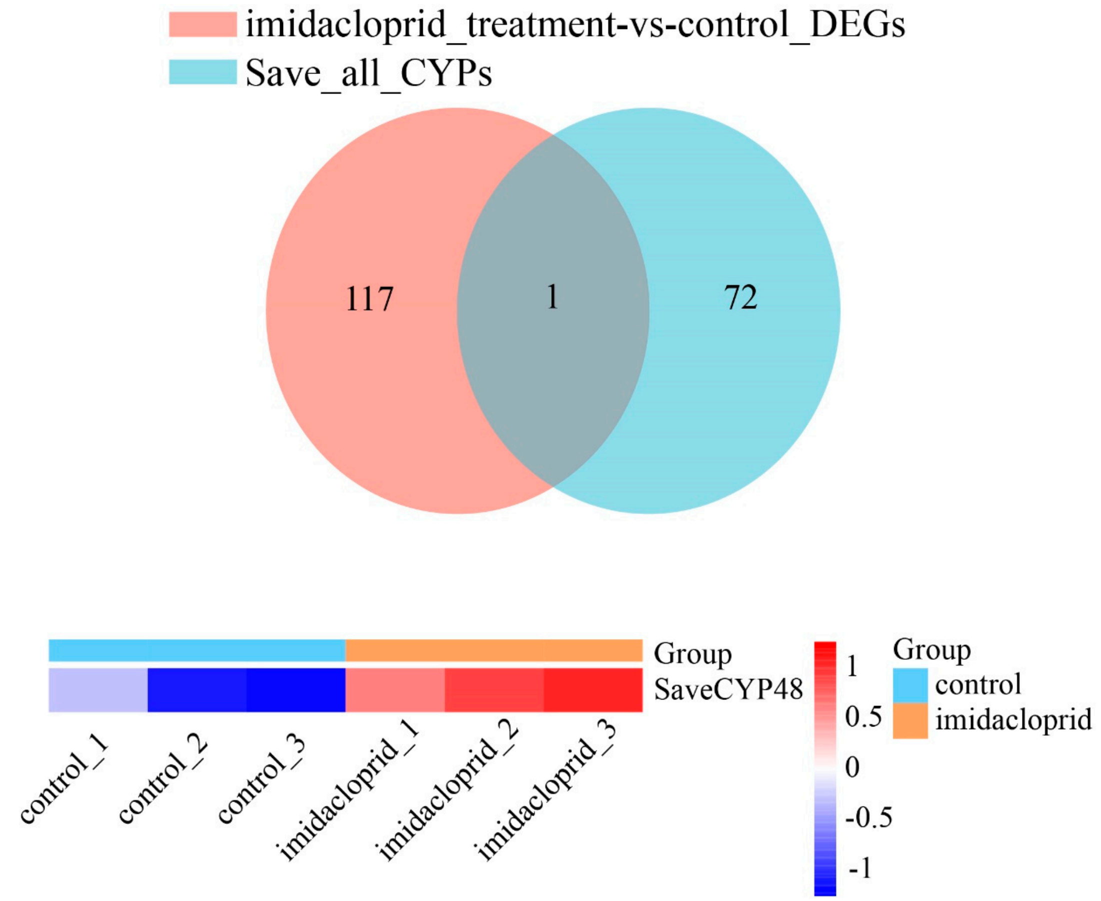

Figure S13. Differentially expressed *CYP450* genes of *Sitobion avenae* aphids fed on wheat treated by imidacloprid.
